# Mechanisms of immunotherapy resistance in small cell lung cancer

**DOI:** 10.20517/cdr.2024.154

**Published:** 2024-12-28

**Authors:** Yunan Nie, Kurt A. Schalper, Anne Chiang

**Affiliations:** ^1^Department of Medical Oncology, Yale School of Medicine, New Haven, CT 06510, USA.; ^2^Department of Pathology, Yale School of Medicine, New Haven, CT 06510, USA.

**Keywords:** Immunotherapy resistance, small cell lung cancer, biomarker, primary resistance, acquired resistance

## Abstract

Small-cell lung cancer (SCLC) is an aggressive neuroendocrine tumor with a poor prognosis. Although the addition of immunotherapy to chemotherapy has modestly improved outcomes, most patients rapidly develop resistance. Resistance to immunotherapy can be broadly categorized into primary resistance and acquired resistance, as proposed by the Society for Immunotherapy of Cancer (SITC) consensus definition. Primary resistance occurs in the setting of failure to respond to immune checkpoint inhibitors (ICIs), while acquired resistance develops after initial response. The mechanisms of acquired and primary resistance to ICI are not well understood in SCLC, denoting an area of critical unmet need.

Both intrinsic and extrinsic mechanisms play significant roles in immunotherapy resistance. Intrinsic mechanisms include defects in antigen presentation, mutations in key genes, reduced tumor immunogenicity, and epigenetic alterations. Extrinsic mechanisms involve the tumor microenvironment (TME), which is a complex interplay of both tumor- and immunosuppressive immune cells, vasculature, and microbiome.

An understanding of these resistance mechanisms is crucial for developing novel therapeutic strategies to advance effective immunotherapy in patients with SCLC, a critical area of unmet need.

## INTRODUCTION

Lung cancer remains the leading cause of cancer-related deaths worldwide, with an estimated 1.8 to 2 million deaths reported in 2022, a figure projected to exceed 3 million by 2045^[1]^. It is divided into two major histologic subtypes: small cell lung cancer (SCLC) and non-small cell lung cancer (NSCLC), with SCLC comprising approximately 15% of all lung cancer cases^[1,2]^. SCLC is an aggressive high-grade neuroendocrine carcinoma that is initially chemo-sensitive but quickly becomes resistant and refractory even to second-line therapies. With current therapies, most patients with SCLC have an expected survival on the order of months, with a 5-year overall survival (OS) of 2%-4.5%^[3]^. While the addition of immunotherapy has improved outcomes, the lack of predictive biomarkers and an incomplete understanding of mechanisms of resistance highlight a need for further translational work.

Patients are commonly classified as having *limited*-stage SCLC (LS-SCLC), where involved areas are confined to the primary site and regional lymph nodes with the possibility of definitive therapy, and *extensive*-stage SCLC (ES-SCLC), where the disease is advanced and treated systemically with palliative intent^[4]^. The American Joint Committee on Cancer (AJCC) Staging System may also be used for SCLC staging to provide more information. SCLC is strongly associated with smoking, with approximately 85% of patients diagnosed with SCLC reporting a history of smoking^[5,6]^. While the incidence among non-smokers varies by region, there appears to be a greater proportion in East Asia^[5]^.

Outcomes in SCLC have historically been and remain poor, with a median OS of 25 to 30 months for LS-SCLC and only 8 to 15 months for ES-SCLC^[7-9]^. For decades, the mainstay of therapy has been concurrent chemoradiotherapy (cCRT) for limited-stage disease and chemotherapy for extensive-stage disease with few options after progression^[10]^. Systemic treatment options for SCLC have been historically sparse, with topotecan approved in 1996 and no new drug approvals until 2019, with the approval of atezolizumab with chemotherapy as first-line therapy followed by durvalumab with chemotherapy in the first-line setting in 2020 and lurbinectedin as second-line therapy in 2020^[11]^. The last five years have seen more new drug approvals than in the 20 years prior due to the advent of immune checkpoint inhibitors (ICIs) and, most recently, tarlatamab, a bispecific T cell engager^[12]^.

Several landmark trials of chemoimmunotherapy in ES-SCLC have been published within the last decade, with practice-changing results. In CASPIAN, durvalumab with or without tremilimumab plus platinum-etoposide was compared to platinum-etoposide, with significant improvements in OS with a hazard ratio (HR) of 0.71 and median OS of 12.9 months versus 10.5 months in the most recent 3-year OS update^[13]^. IMPower133 demonstrated that the addition of atezolizumab to first-line platinum-etoposide also conferred a survival benefit, with a HR of 0.76 and median OS of 12.3 *vs.* 10.3 months favoring the chemoimmunotherapy regimen^[14,15]^. These trials led to the approval of durvalumab or atezolizumab in combination with carboplatin and etoposide as frontline standard of care therapy for ES-SCLC.

While the addition of immunotherapy to chemotherapy in the first-line setting has clearly improved survival and clinical outcomes, median progression-free survival (PFS) and OS remain under 6 months and 13 months, respectively^[13-21]^. Other trials of immune checkpoint inhibitors (ICIs) in later-line therapy in SCLC include CheckMate-032, a phase 1/2 multi-arm trial that assessed nivolumab, a PD-1 inhibitor, with and without the addition of ipilimumab, a CTLA-4 inhibitor, and KEYNOTE 158, a phase 2 study of pembrolizumab, a PD-1 inhibitor, as third-line therapy^[19,22]^. These trials led to the United States Food and Drug Association (FDA)’s accelerated approval of nivolumab in 2018 and pembrolizumab in 2019 as a third-line treatment after progression on a platinum-containing chemotherapy regimen and at least one other line of treatment^[23]^. Pembrolizumab and nivolumab were withdrawn from the market for the treatment of SCLC as the Phase III trials CheckMate 451, CheckMate 331, and KEYNOTE 604 did not meet their primary endpoints of OS, although they did demonstrate a significant improvement in PFS, a secondary endpoint^[24-26]^.

More recently, the first interim analysis of ADRIATIC, a Phase 3 trial of consolidation durvalumab compared to placebo after cCRT for LS-SCLC, demonstrated a PFS and OS benefit, with a HR of 0.73 for death and 55.9 months *vs.* 33.4 months for OS^[27]^. Patients were treated with up to 24 months of consolidation durvalumab after completion of cCRT. PFS was also significantly longer at 16.6 months *vs.* 9.2 months, with a HR for progression or death of 0.76. Consolidation durvalumab after cCRT is now included in the National Comprehensive Cancer Network (NCCN) guidelines for LS-SCLC and is the new standard of care. A summary of major trials of immunotherapy in SCLC is available in Table 1.

**Table 1:** Selected key trials of immunotherapy in LS-SCLC and ES-SCLC

**Study**	**No.**	**Phase/Setting**	**Authors**	**Year of publication**		**Immune checkpoint inhibitor regimen**	**Comparison arm**	**Primary endpoint**	**Results**		**FDA approved?**	**NCCN guidelines inclusion**
First line												
IMPower133	NCT02763579	Phase 1/3, multinational	Horn *et al.*^[14]^; Liu *et al.*^[15]^ (updated results)	2018, 2021 (updated results)		Atezolizumab + EP	EP + placebo	PFS, OS	Atezolizumab + EP significantly improved PFS (HR 0.77; 95% CI 0.62-0.96; P = 0.02) and OS (HR 0.76; 95% CI 0.60-0.95, P = 0.0154)		Yes	Yes
CASPIAN	NCT03043872	Phase 3, multinational	Paz-Ares *et al.*^[13,16]^	2019, 2022 (updated results)		Durvalumab (+/- tremilimumab) + EP	EP + placebo	OS	Durvalumab + EP significantly improved OS (HR 0.71, 95% CI 0.60-0.86, P = 0.0003)		Yes	Yes
ASTRUM-005	NCT04063163	Phase 3, multinational	Cheng *et al.*^[17]^	2022		Serplulimab + EP	EP + placebo	OS	Serplulimab + EP significantly improved OS (HR 0.63, 95% CI 0.49-0.82, P < 0.001)		No; NMPA approved	No
CAPSTONE-1	N/A	Phase 3, national	Wang *et al.*^[28]^	2022		Adebrelimab + EP	EP + placebo	OS	Adebrelimab + EP significantly improved OS (HR 0.72, 95% CI 0.58-0.90, P = 0.0017)		No; NMPA approved	No
ETER701	N/A	Phase 3, national	Cheng *et al.*^[29]^	2024		Benmelstobart + anolitinib + EP	EP + double placebo; EP + anlotinib + placebo	PFS, OS	Benmelstobart + anlotinib + EP significantly improved OS (HR 0.61, 95% CI 0.45-0.79, P = 0.0003) and PFS (0.32, 95% CI 0.26-0.41, P < 0.0001)		No	No
KEYNOTE 604	NCT03066778	Phase 3, multinational	Rudin *et al.*^[26]^	2020		Pembrolizumab + EP	EP + placebo	PFS, OS	Pembrolizumab + EP significantly improved PFS (HR 0.75, 95% CI 0.61-0.91, P = 0.0023), significance threshold not met for OS (HR 0.80, 95% CI 0.64-0.98, P = 0.0164)		No	No
CheckMate 451	NCT02538666	Phase 3, multinational	Owonikoko *et al.*^[24]^	2021		Nivolumab +/- ipilimumab maintenance after EP	EP + placebo maintenance	OS	Primary endpoint not reached		No	No
CA184-156	NCT01450761	Phase 3, multinational	Reck *et al.*^[18]^	2016		Ipilimumab + EP	EP + placebo	OS	Primary endpoint not reached		No	No
Relapsed/Refractory												
CheckMate 331	NCT02481830	Phase 3, multinational	Spigel *et al.*^[25]^	2021		Nivolumab	Topotecan or amrubicin	OS	Primary endpoint not reached		No (marketing authorization withdrawn)	Yes
CheckMate 032	NCT01928394	Phase 1/2, multinational	Antonia *et al.*^[19]^; Ready *et al.*^[21]^ (updated results)	2016, 2020 (updated results)		Nivolumab +/- ipilumumab	N/A	ORR	ORR higher in nivolumab + ipilumumab group (odds ratio 2.12, 95% CI 1.06-4.25, P = 0.03), OS was similar			
KEYNOTE 158	NCT02628067	Phase 2, multinational	Chung *et al.*^[22]^	ASCO 2018 Abstract	2020 (pooled analysis of KEYNOTE 158 and phase 1b KEYNOTE 028)	Pembrolizumab	N/A	ORR	ORR 18.7% overall	Pooled analysis of KEYNOTE-028 and KEYNOTE-158 with ORR 19.3%, 95% CI 11.4-29.4, with 61% of responders with responses > 18 months		Yes
KEYNOTE 028	NCT02054806	Phase 1b, multinational	Ott *et al.*^[20]^; Chung *et al.*^[22]^	2017		Pembrolizumab	N/A	Safety, tolerability, ORR	Safety consistent with known safety profile of pembrolizumab, ORR 33%, 95% CI 16%-55%			
Limited stage												
STIMULI	NCT02046733	Phase 2, multinational	Peters *et al.*^[10]^	2022		Ipilimumab/nivolumab consolidation after cCRT	Placebo	PFS	Closed early due to slow accrual		No	No
ADRIATIC	NCT03703297	Phase 3, multinational	Cheng *et al.*^[27]^	2024		Durvalumab +/- tremilimumab consolidation after cCRT	Placebo	PFS, OS	Durvalumab consolidation significantly improved PFS (HR 0.76, 95% CI 0.59-0.98, P = 0.02) and OS (HR 0.73, 95% 0.54-0.98, P = 0.01)		TBD	Yes

No: ClinicalTrials.gov identifier; OS: Overall survival; PFS: Progression free survival; EP: Platinum-etoposide; cCRT: Concurrent chemoradiotherapy; ORR: Objective response rate; SCLC: Small cell lung cancer; LS-SCLC: *Limited*-stage SCLC; ES-SCLC: *Extensive*-stage SCLC; HR: Hazard ratio; NCCN: National comprehensive cancer network.

There is significant variation in the duration and depth of response among patients with SCLC who receive immunotherapy, and the mechanism by which patients develop primary or acquired resistance to therapy is poorly understood. It is likely that heterogeneity within SCLC, driven by genomic and transcriptomic variation, is a driver of this differential response. As most patients with SCLC are diagnosed with advanced disease, surgical resections are rare and limited tissue availability has historically been a barrier for translational work^[30]^. In this review, we will discuss the clinical definitions of immunotherapy resistance and mechanisms of resistance to immunotherapy in patients with SCLC. As the molecular mechanisms of immunotherapy resistance in SCLC are understudied, we will review the evidence in SCLC in the context of mechanisms of resistance extrapolated from more extensively studied tumor types, such as NSCLC and melanoma.

## DEFINITION OF IMMUNOTHERAPY RESISTANCE

A significant challenge in studying immunotherapy resistance, in general, is the lack of a uniform definition, which is made especially difficult in the context of combination therapies where immunotherapy is paired with other agents. The Society for Immunotherapy of Cancer (SITC) has published consensus definitions for resistance to single-agent immunotherapy, combination immunotherapies, chemoimmunotherapy, and adjuvant immunotherapy^[31,32]^. In general, while the taskforce felt 6-8 weeks (or 2 cycles) of the immunotherapy component was likely necessary in most circumstances, they also included the caveat that resistance may be determined sooner if there is clear rapidly progressing disease.

Here, we explore how SITC immunotherapy resistance definitions can be potentially applied to small-cell lung cancer, which has not previously been clearly defined.

### Immunotherapy monotherapy resistance in ES-SCLC

**Figure 1:**
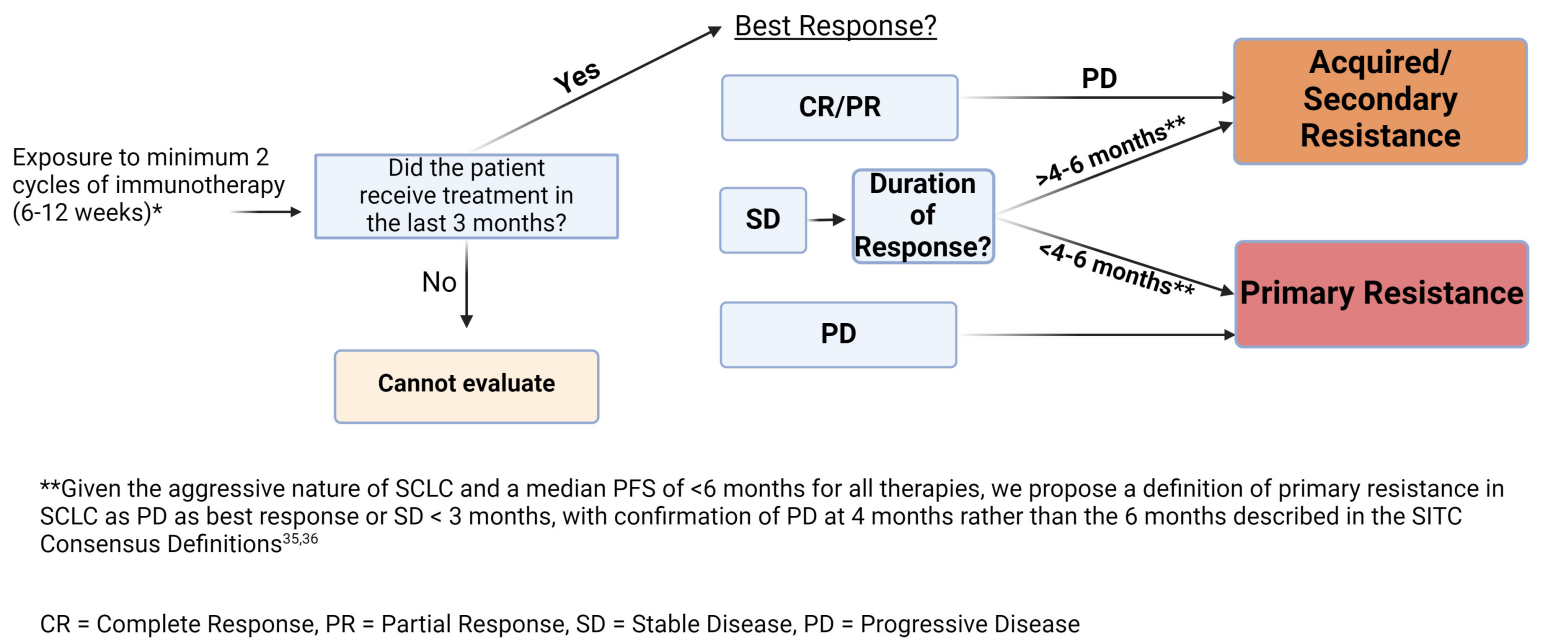
Immunotherapy monotherapy resistance in ES-SCLC. Schema of SITC immunotherapy monotherapy definitions, with a shorter timeframe for consideration of acquired resistance for ES-SCLC. SCLC: Small cell lung cancer; ES-SCLC: *Extensive*-stage; SITC: Society for immunotherapy of cancer.

In patients who receive single-agent immunotherapy, primary resistance was defined by SITC as disease progression after two cycles or at least 6 weeks of treatment and no more than 6 months of exposure to ICIs^[31,32]^. The patient must have received a dose of ICI within 12 weeks (or 3 months) of the evaluation for resistance [Figure 1]. Secondary or acquired resistance was defined as progression after a documented, confirmed objective response or prolonged stable disease for ≥ 6 months, independent of target lesion measurements. The patients were separated into adequate *vs.* inadequate exposure groups, with progression within 6-12 weeks after their last dose versus > 12 weeks after their last dose, respectively. An alternate classification of “early relapse” *vs.* “late relapse” was also proposed.

In ES-SCLC, trials of single-agent immunotherapy include CheckMate 331 with nivolumab monotherapy, CheckMate 032 with a nivolumab monotherapy arm, and KEYNOTE 158 and 028 with pembrolizumab in relapsed/refractory disease [Table 1]. CheckMate 331 and CheckMate 032 demonstrated a median PFS of 1.4 months and 1.4 months, respectively^[21,25]^. The duration of response (DOR), defined as the time from the first confirmed complete response (CR) or partial response (PR) to documented progression, was 8.3 months (7.0-12.6) and 15.8 months (7.1-NR) in CheckMate 331 and CheckMate 032, respectively. The combined analysis of KEYNOTE 158 and KEYNOTE 028 demonstrated a median PFS of 2.0 months, with a DOR of 25.9 months (4.1-35.8)^[22]^. Although the median PFS was 2 months or less for the immunotherapy arm in these trials, the duration of response in the patients who did respond ranged from 4.1 months to not reached (NR), indicating that patients with a response had a longer PFS than would be suggested by the median PFS among all patients. In addition, the SITC discusses that for aggressive malignancies, “a shorter period of disease control may still indicate clinical benefit, and, as such, the consensus of the group was that a shorter duration of best response should be required for the definition for secondary resistance to ICI combinations for patient safety concerns”^[31]^. In light of these data, we suggest that the cut-off between primary and secondary resistance in single-agent immunotherapy for ES-SCLC also be set at a shorter time point than the 6 months published in the SITC consensus definitions, e.g., 4-6 months, as displayed in Figure 1.

### Combination immunotherapy resistance in ES-SCLC

For combination ICI regimens, the taskforce recommended a confirmatory scan 4 weeks after progressive disease (PD), with primary resistance defined as a minimum of exposure to 2 cycles of both drugs (6-12 weeks) with best response PD or stable disease (SD) < 6 months. Similarly, secondary resistance was defined as progression after initial objective response or durable stable disease after > 6 months of drug exposure^[31,32]^. If induction dosing was utilized, where two ICIs were administered for a limited number of cycles followed by monotherapy maintenance, PD occurring after 6 months was defined as secondary resistance to the monotherapy for the drug that was continued. In CheckMate 032 which included ipilimumab/nivolumab arms, median PFS was 1.5 months with DOR of 10 months (6.7-NR)^[21]^. Given this PFS, we feel that it would again be reasonable to set the cut-off shorter than 6 months for primary versus secondary resistance in SCLC.

### Chemoimmunotherapy resistance in ES-SCLC

Defining immunotherapy resistance in combined chemoimmunotherapy regimens poses a significant challenge due to the difficulty in determining the role of the ICI versus the chemotherapy in treatment response. In the case of chemoimmunotherapy combinations in the SITC taskforce consensus, primary resistance was defined as progression within the first 6 months of therapy, irrespective of the initial response^[31]^. Secondary or acquired resistance to chemoimmunotherapy was not defined due to the impossibility of establishing the contributions of the individual components.

**Figure 2:**
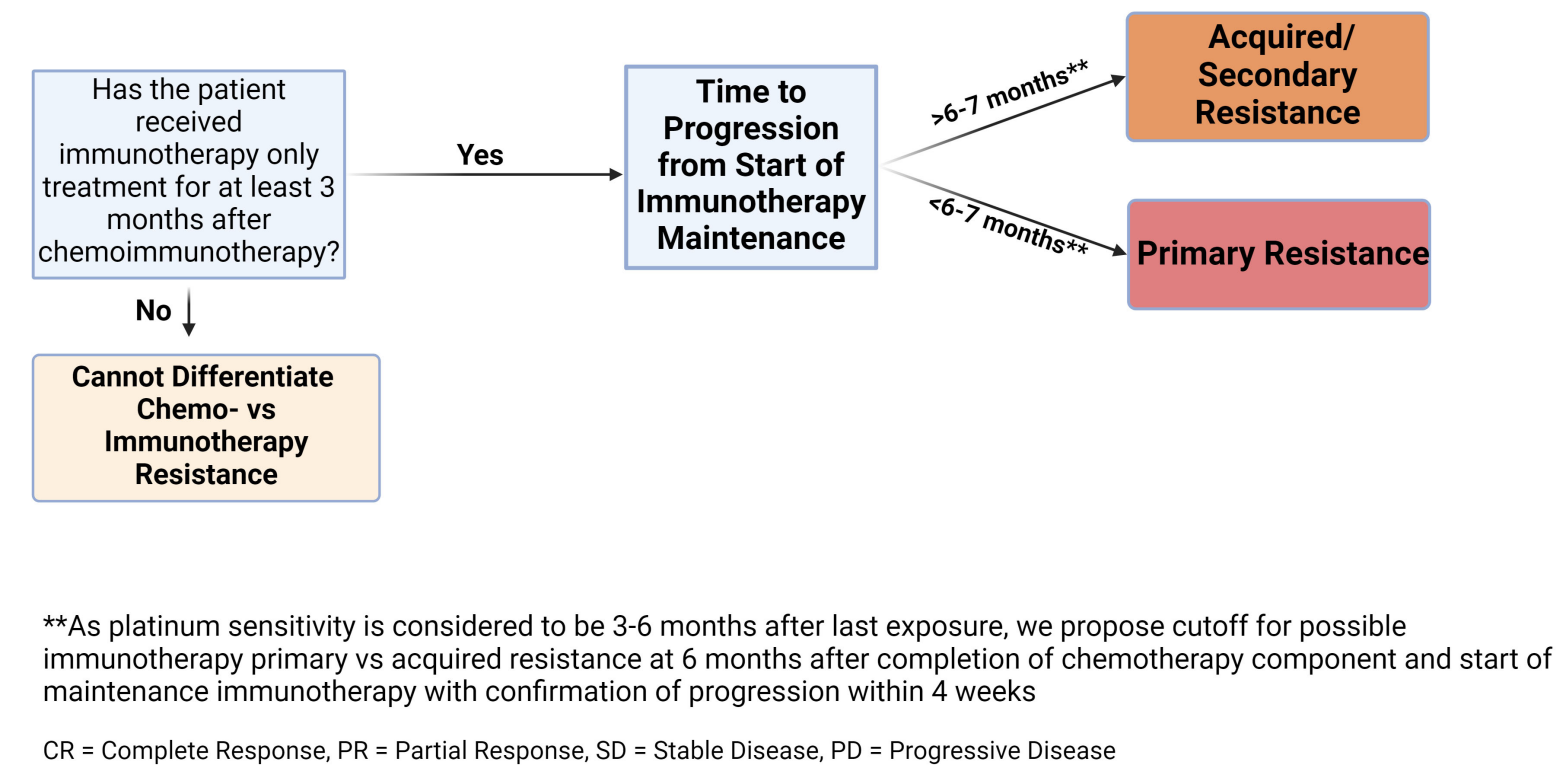
Chemoimmunotherapy resistance in ES-SCLC. Schema of SITC chemoimmunotherapy definition with a potential shorter cut-off for primary versus acquired resistance in ES-SCLC. SCLC: Small cell lung cancer; ES-SCLC: *Extensive*-stage; SITC: Society for immunotherapy of cancer.

Notably, patients with ES-SCLC are treated with a combination of chemoimmunotherapy and the vast majority of SCLC are platinum-sensitive at the initiation of therapy. Per NCCN guidelines, a chemotherapy-free interval (CTFI) of 3-6 months is used to determine candidacy for platinum rechallenge and patients who respond to rechallenge are considered platinum-sensitive^[33]^. Thus, if there is relapse/progression within 3 months of completing chemotherapy, it is impossible to determine primary ICI *vs.* chemotherapy resistance in ES-SCLC due to the definition of platinum sensitivity [Figure 2]. If there is progression within 6 months of starting maintenance immunotherapy, this may represent primary resistance to the immunotherapy component. Acquired resistance, therefore, could be considered if progression occurs after 6 months post-initiation of maintenance immunotherapy, but it remains difficult to parse out the contribution of the components without platinum rechallenge.

### Adjuvant/consolidation immunotherapy resistance in LS-SCLC

With the inclusion of consolidation durvalumab after cCRT in the NCCN guidelines v2.2025, the definition of adjuvant/consolidation immunotherapy resistance will need to be addressed^[33]^. In the adjuvant setting, primary resistance was defined as recurrence ≤ 12 weeks after completion/discontinuation of therapy or recurrence on therapy by SITC. For patients who progress after stopping therapy for any multitude of reasons, tumor regrowth ≤ 12 weeks after therapy was halted was deemed primary resistance. If recurrence/regrowth occurred after 12 weeks after treatment discontinuation, this could not be determined as resistance.

cCRT is the standard of care for LS-SCLC not amenable to surgical resection. The vast preponderance of patients receive both chemotherapy and radiation, which can make determination of resistance difficult^[33]^. In the CONVERT trial, which evaluated once-daily versus twice-daily radiation with concurrent chemotherapy, median PFS was 14.3 months and 15.4 months, respectively^[34]^. In STIMULI, a trial of consolidation ipilimumab/nivolumab that was closed early due to slow accrual, the median PFS in the control arm without immunotherapy was 14.5 months^[10]^. In ADRIATIC, the control arm demonstrated a median PFS of 9.2 months versus 16.6 months in the durvalumab arm which conferred a 7.4 month PFS benefit^[27]^. 46.0% of patients progressed and discontinued treatment before completing 2 years of treatment, with patients receiving a median of 12.9 infusions of durvalumab. Interestingly, the PFS in the control arm of STIMULI was notably longer than in the control arm of ADRIATIC. This difference may be due to the smaller study size of STIMULI compared to ADRIATIC, or variation in patient recruitment as ADRIATIC recruited from a global population while STIMULI recruited in Europe. Furthermore, while there was a higher proportion of patients in STIMULI who received once daily rather than twice daily Radiation Therapy (RT) than in ADRIATIC, the CONVERT trial, which addressed once-daily versus twice-daily RT, did not find a significant difference in outcomes.

**Figure 3:**
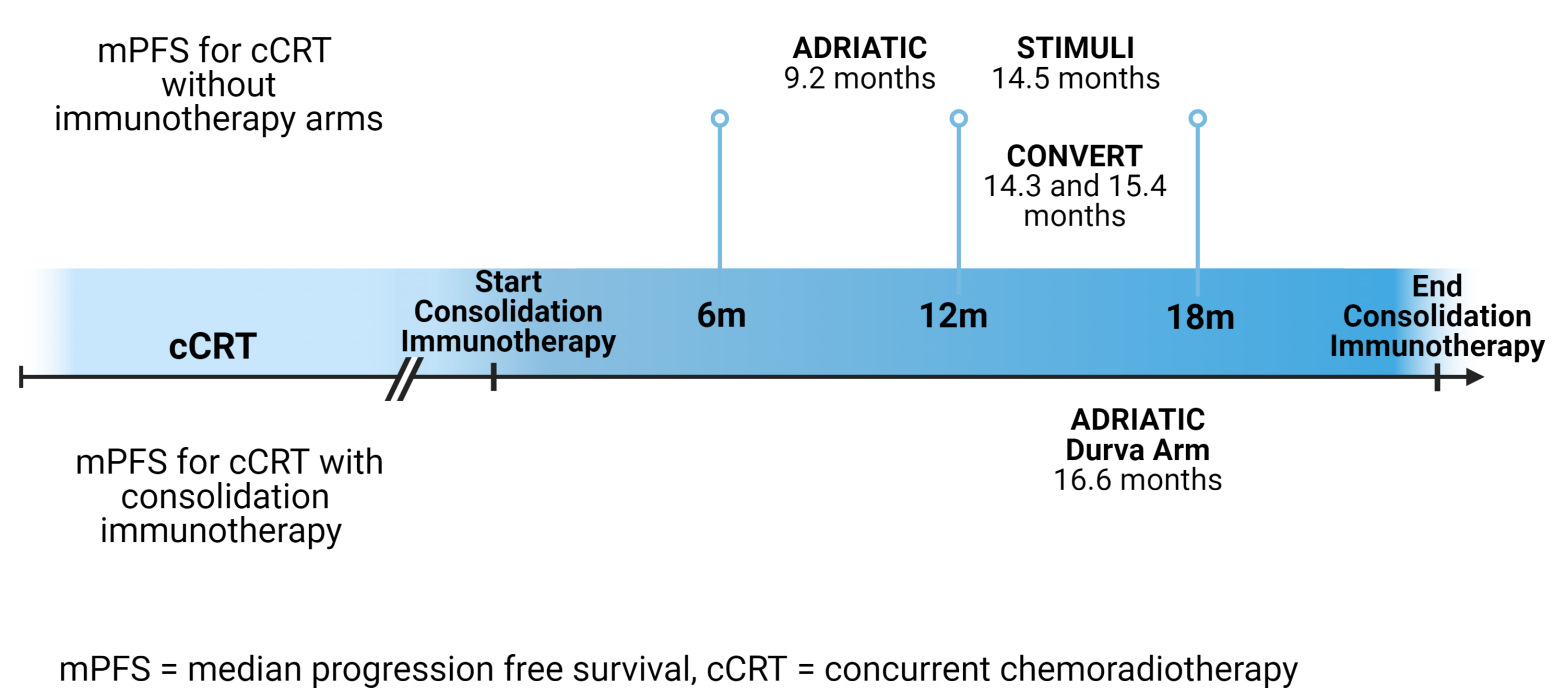
We summarize the median progression-free survival for cCRT and cCRT with consolidation immunotherapy through a timeline of median PFS after cCRT without consolidation immunotherapy *vs.* median PFS after cCRT with consolidation durvalumab as described in ADRIATIC^[27]^. cCRT: Concurrent chemoradiotherapy; PFS: Progression-free survival.

Given these time points [Figure 3], it is not possible to determine the contribution of radiation, chemotherapy, or immunotherapy resistance if there is recurrence within 1 year of starting durvalumab consolidation. As almost half of patients progressed prior to completion of the 2 years of consolidation treatment and patients received a median of 12.9 infusions, it would also be difficult to define primary resistance as within 12 weeks of completing consolidation/adjuvant therapy. More data are needed to delineate primary versus acquired resistance in this population.

## MECHANISMS OF IMMUNOTHERAPY RESISTANCE IN SCLC

### Heterogeneity in SCLC

To frame the discussion of immunotherapy resistance mechanisms in SCLC and provide a plausible explanation for the significant variation in initial response and primary resistance, a brief discussion of SCLC genomic and transcriptional heterogeneity is essential. While SCLC has historically been treated as a homogenous disease, SCLC classification by both genomic and transcriptomic markers and the subsequent recognition of heterogeneity within this disease has emerged as a promising explanation for variability in immunotherapy response.

Genomically, the loss of the tumor suppressor genes *RB1* and *TP53* by deleterious mutations or deletions is near universal in SCLC^[35]^. A recent study, however, of twenty cases of *RB1* and *TP53* wild-type SCLC demonstrated that this subset of aggressive tumors exhibited genomic and pathologic features of pulmonary carcinoids and were associated with chromothripsis-massive, localized chromosome shattering^[36]^. This suggests that a small subset of SCLC may be due to transformation from carcinoids, a low-grade neuroendocrine tumor (NET), and may be differentially responsive to immunotherapy^[37]^. Mutations on other genes have been reported, including *ARID1A* and *ARID1B, CREBBP,* and *FGFR* amplification, as well as genes in the PIK3/mammalian target of rapamycin (mTOR) pathway (loss of phosphatase and tensin homolog (PTEN), rapamycin-insensitive companion of mTOR (RICTOR) amplification, regulatory associated protein of MTOR complex 1 (RPTOR), tuberous sclerosis complex 2 (TSC2)) that contribute to heterogeneity within this disease^[38,39]^.

Transcriptomic subtypes have also emerged to support heterogeneity in SCLC, and are associated with differential response to immunotherapy. At least four SCLC subtypes, SCLC-A, SCLC-N, SCLC-P, and SCLC-I, have been described based on comprehensive transcriptional profiles^[40,41]^. These subtypes show prognostic associations and are based on the expression of three major transcription factors: achaete-scute homolog 1 (ASCL1), neurogenic differentiation factor 1 (NEUROD1), and POU class 2 homeobox 3 (POU2F3) in combination with the presence of a proinflammatory gene signature^[40,41]^. SCLC-A and SCLC-N are characterized by the expression of ASCL1 and NEUROD1, respectively, which are involved in neuroendocrine differentiation and characterized as “neuroendocrine subtypes.” SCLC-P is considered a “non-neuroendocrine subtype” as it is characterized by POU2F3 expression, which drives the expression of tuft cells in the mucosal epithelium^[41]^. SCLC-I is characterized by low levels of the transcriptional factors characteristic of the other three subtypes and demonstrates an inflammatory transcriptional profile. A subtype referred to as SCLC-Y has also previously been reported in the setting of elevated Yes-associated protein 1 (YAP1), which is sometimes described as a fourth subtype instead of SCLC-I^[40,42]^. A recent study identified further division of SCLC-I into SCLC-I-neuroendocrine (SCLC-I-NE) and SCLC-I-non-neuroendocrine (SCLC-I-nonNE) phenotypes^[43]^. Specific subtypes of SCLC, such as SCLC-I, may derive greater benefit from ICIs, indicating varied resistance mechanisms^[43]^. Non-neuroendocrine tumors have demonstrated significantly higher CD45+ leucocytes with increased CD8+ effector tumor-infiltrating lymphocytes (TILs) and higher MHC I expression, which likely contribute to variations in immunotherapy response^[44-47]^. For example, SCLC-I-NE, which was characterized by low tumor-associated macrophage (TAM) and high T-effector signal, displayed better clinical outcomes with atezolizumab and chemotherapy treatment^[43]^.

Despite marked progress in the understanding of the molecular basis of SCLC, a universally accepted molecular classification is evolving. The clinical significance and therapeutic value of the reported genomic and transcriptomic subtypes are under investigation.

### Overview of mechanisms of immunotherapy resistance

Broadly speaking, mechanisms of resistance can be characterized as tumor-intrinsic or tumor-extrinsic^[48]^. Tumor-intrinsic mechanisms are inherent to the malignant cells and include changes in the antigen presentation machinery or in the cancer cell immunogenicity. Tumor-extrinsic mechanisms, on the other hand, include the effect of other cell types within the tumor microenvironment (TME) or of other mechanisms outside the tumor bed. As the mechanisms of immunotherapy response and resistance in SCLC are relatively understudied, we will discuss SCLC-specific evidence in the context of broader research from other better-characterized solid tumors.

**Figure 4:**
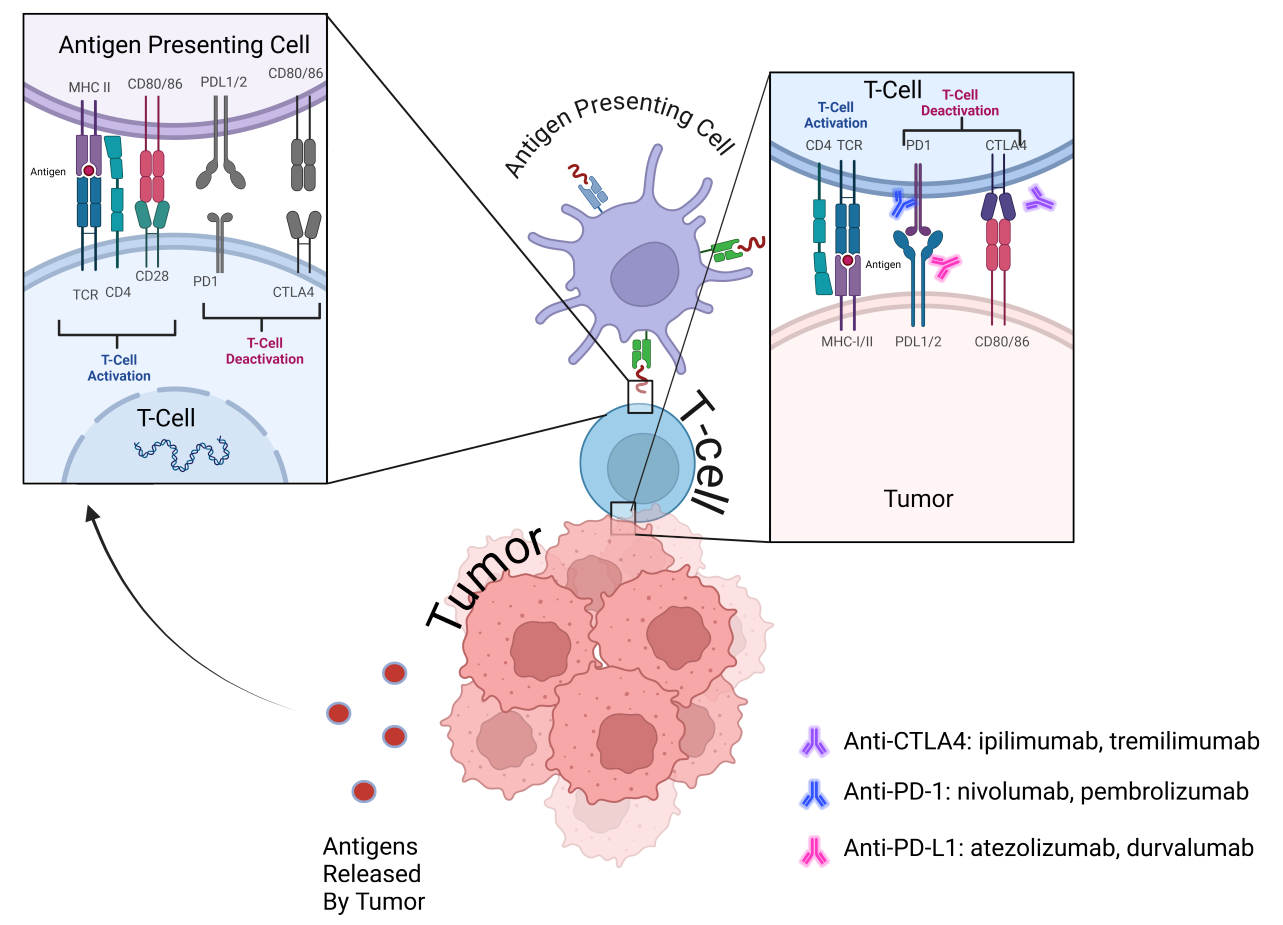
Representation of T cell activation in antitumor response and deactivation through immune checkpoints. Antigen-presenting cells (APCs) process and package tumor antigens into peptides to be displayed on its surface with major histocompatibility complex (MHC) molecules to T cells, accompanied by costimulatory signal binding of CD28 and CD80/86 to promote T cell activation. In addition, the presentation of tumor surface antigens via MHC activates T cell response and expansion. The binding of PD-L1/2 to PD-1 and CD80/86 to CTLA4 suppresses the antitumor immune response, allowing immune evasion by tumor cells. Blockade of these immune checkpoints through ICI restores T cell activity and reactivates the antitumor response. ICI: Immune checkpoint inhibitor.

The antitumor response is dependent on effector T cell activation, which requires both an interaction between the T cell receptor (TCR) and surface antigens in malignant cells presented by the major histocompatibility complexes (MHC) and ligation of CD28, a T cell costimulatory receptor, to CD80 or CD86 [Figure 4]^[49]^. Upon TCR engagement, phosphorylation of intracellular tyrosine residues leads to the recruitment of phosphatidylinositol 3-kinase (PI3K) and promotes activation of nuclear factor-kappa B (NF-kB) and protein kinase B/Ak strain transforming (PKB/AKT) to maintain T cell survival and activation. Tumor-specific naïve CD8+ T cells then differentiate into effector T cells and undergo clonal expansion to ultimately kill tumor cells expressing their cognate antigens. Co-inhibitory receptors such as programmed cell death 1 (PD-1) and cytotoxic T lymphocyte antigen 4 (CTLA-4) are expressed on most activated T cells to limit unchecked activation and prevent tissue damage but can also be co-opted into immune evasion by the tumor. Blockade of these immune checkpoint pathways through ICIs forms the foundation of the immunotherapy response. Alterations or deficiencies in any part of the above pathways can lead to immune evasion and immunotherapy resistance.

APCs process and package tumor antigens into peptides to be displayed on its surface with MHC molecules to T cells, accompanied by costimulatory signal binding of CD28 and CD80/86 to promote T cell activation. In addition, the presentation of tumor surface antigens via MHC activates T cell response and expansion. The binding of PD-L1/2 to PD-1 and CD80/86 to CTLA4 suppresses the antitumor immune response, allowing immune evasion by tumor cells. Blockade of these immune checkpoints through ICI restores T cell activity and reactivates the antitumor response.

### Intrinsic mechanisms of immunotherapy resistance

#### Tumor immunogenicity and antigen presentation

The availability of tumor neoantigens is expected to be a key feature in the immunogenicity of cancer cells and for activation of the adaptive immune response. Thus, low tumor mutation burden (TMB) is associated with decreased neoantigen load and decreased tumor immunogenicity, which is associated with limited immunotherapy responses in NSCLC^[50]^. Conversely, a high TMB is generally associated with increased sensitivity to ICIs. However, TMB is not an accurate predictor of immunotherapy benefits and could be affected by multiple factors, such as challenges in identifying optimal cut-points, differences across genomic testing methods and the impact of intratumor genetic heterogeneity and clonal diversity of malignant cells^[51]^.

PD-L1 expression has been associated with clinical benefit to ICIs and outcomes can be stratified by PD-L1 expression in multiple solid tumors, including head and neck squamous cell cancers, non-small cell lung cancers, and breast carcinomas. Prevalence of PD-L1 expression in SCLC, however, is prominently lower at baseline, with one study finding less than 20% of the patients with positive expression as compared to NSCLC, where PD-L1 prevalence is much higher at 52%-67%^[52-55]^. Moreover, PD-L1 expression in SCLC does not consistently correlate with response to ICIs. Studies using small cohorts of SCLC have also found low expression of CTLA-4, T-cell immunoglobulin and ITIM domain (TIGIT), and other immunosuppressive molecules commonly targeted by ICIs, with increased expression of alternate checkpoints CD276 and CD200^[56]^. Another potential target for immunotherapy is B7-H3, a member of the same B7 ligand family as PD-L1, which has been found to be highly expressed in SCLC, with B7-H3 expressed in 64.9% of SCLC cases compared to PD-L1 in 7.3% of cases^[57]^. TMB, a commonly used marker of tumor immunogenicity, varies in SCLC, ranging from 0 to 276.3 mut/Mb in one large cohort with a median of 7.8 mut/Mb. Another study reported a median TMB of 12.7 mut/Mb^[58]^. The association of TMB with survival after ICIs has been mixed in SCLC, with findings of a significant association between high TMB and improved outcomes in the CheckMate 032 cohort but not in the IMPower 133 cohort, albeit using blood TMB^[43,59]^. These results suggest that a fraction of SCLC display relatively low TMB and, therefore, limited neoantigens that can be recognized as non-self and mediate robust antitumor adaptive immune rejection.

The antigen presentation machinery is essential for the antitumor immune response. Multiple studies suggest that loss of MHC I presentation via genomic alterations or protein dysregulation, or expression of alternate MHC class IB molecules, such as human leukocyte antigen (HLA)-E and HLA-G, is associated with immune evasion and immunotherapy resistance across multiple solid tumors^[60,61]^. transporter associated with antigen processing (TAP), a peptide transporter that transfers proteasome and immunoproteasome peptide into the endoplasmic reticulum, is composed of two subunits, TAP 1 and TAP2. These peptides are then loaded onto newly assembled MHC I molecules^[62,63]^. Calreticulin, TAP-binding protein related (TAPBPR), endoplasmic reticulum aminopeptidase (ERAP) 1 and 2, endoplasmic reticulum protein 57 (ERP57) are components of the peptide loading complex, which are essential for the stabilization of MHC I peptide complexes. Mutations or downregulation of these pathways could result in immune evasion through decreased antigen presentation. Epigenetic silencing of TAP1 by EZH2 has been associated with low MHC I antigen presentation. β2 microglobulin (B2M) expression is well characterized as essential for MHC I expression and function. Deficiency of β2M by deleterious mutations or silencing can lead to defects in the antigen presentation mechanism and has been associated with resistance across multiple solid tumors^[60]^. Surface expression of MHC I is stimulated by interferons, particularly IFNγ (Type II IFN), which is secreted with the activation of the JAK/STAT pathway^[64,65]^. In a study of melanoma with acquired resistance to ICI, JAK1-mutated melanoma cell lines were resistant to both Type I and II IFN signaling, while JAK2-mutated cells were resistant to Type II IFN but responded to Type I IFN signaling. Mutations or loss of the components of this signaling cascade, particularly JAK1/JAK2, therefore, are associated with resistance^[66]^.

Suppressed antigen presentation with low β2M and HLA I expression is seen more frequently in SCLC than in other lung malignancies, despite some of these tumors demonstrating high TMB and C:G to A:T transversions^[47,67,68]^. MHC I and MHC II expression are significantly lower in SCLC as compared to NSCLC, potentially leading to decreased CD8+ and CD4+ T cell activation^[56,69]^. DLL3, which is highly expressed in SCLC, can suppress the stimulator of IFN genes (STING) pathway and inhibit antigen presentation as well. The association of MHC I with clinical outcomes is supported by evidence that patients with SCLC that expressed low MHC I displayed less intratumoral infiltration of cytotoxic T cells and worse OS with immunotherapy than tumors with high MHC I expression^[45]^. In addition, differential expression of mitochondrial electron transport chain pathways in SCLC can also mediate antigen presentation and T cell-mediated killing^[70,71]^.

#### Mutations and gene expression

Specific gene mutations have been associated with immunotherapy sensitivity and resistance, but a clear driver mutation for immunotherapy resistance has not yet been identified in SCLC. RB1 mutations are the second most frequent alteration in SCLC, and although it is most well-known for its role as a tumor suppressor gene, it is also implicated in immune function and response^[72,73]^. Dowlati *et al.* examined a cohort of SCLC patients who received ICI monotherapy and found that patients with RB1 wild-type tumors had significantly better outcomes^[37,39]^. Using RNAseq, they found that patients with RB1 mutations demonstrated downregulated immune-related genes and an immune exclusion phenotype^[37]^. Two well-known alterations associated with poor immunotherapy outcomes in NSCLC are STK11 gene inactivation and KEAP1 depletion, which are associated with epigenetic inhibition of STING important to the T cell activation pathway and reduced leukocyte infiltration with differential immune cell recruitment, respectively^[74,75]^. Of note, these mutations have also been associated with poor prognosis with chemotherapy and anti-angiogenesis agents, so the effect is not exclusive to immunotherapy and may represent an overall poor prognosis in general^[76]^.

Approximately 3% of cases in one large cohort of SCLC patients had KEAP1 alterations; silencing of this gene has been shown to confer chemoresistance *in vitro* through deregulation of the nuclear factor erythroid 2-related factor 2 (NRF2) and neurogenic locus notch homolog protein 1 (NOTCH) pathways, making this an attractive target for further studies^[77]^. It is unclear, however, if KEAP1 alterations in SCLC confer the same association with decreased immunotherapy response as seen in NSCLC. There was also a subgroup of SCLC with STK11 mutations, though the association with decreased response to ICIs is again unclear. In addition, SCLC has a high prevalence of genetic alterations in the PI3K/AKT/mTOR pathway, found in approximately one-third of patient samples in one study^[78]^. Elevated myelocytomatosis oncogene (MYC) expression is common in SCLC, has been associated with resistance to platinum chemotherapies, and is likely involved in immunotherapy response given evidence that it is involved in IFN signaling^[79]^. Overexpression and deregulation of MYC have been implicated in resistance to immunotherapy in other cancers, including melanoma and head and neck squamous cell carcinoma, through decreased IFNγ via JAK2 downregulation^[80]^. Intrinsic expression of STING is also significantly reduced in SCLC, leading to decreased T cell activation^[81]^.

#### Epigenetic regulators

Alterations in epigenetic regulators such as Enhancer of Zeste Homolog 2 (EZH2) and lysine-specific demethylase 1 (LSD1) have been implicated in downregulation of MHC I and loss of antigen presentation in SCLC^[82-84]^, leading to decreased activation and recognition by the immune system. Epigenetic regulators have been implicated in the response to immunotherapy. EZH2 is an enzymatic subunit of the polycomb repressive complex 2, which catalyzes trimethylation of histone H3 at lysine 27, leading to gene silencing, and LSD1 is a class I demethylase that removes regulatory methyl groups from histone H3 at lysine positions 4 and 9. LSD1 gene expression is encoded by KMD1A, which is highly expressed in SCLC, and correlates with worse survival outcomes for patients treated on CheckMate 032^[84,85]^. Both EZH2 and LSD1 activation have been associated with the downregulation of the antigen presentation machinery in SCLC. Ongoing clinical studies of EZH2 and LSD1 inhibitors demonstrate promise for overcoming immunotherapy resistance.

### Extrinsic mechanisms of immunotherapy resistance

The TME includes the extracellular matrix, vasculature, immune cells, stromal cells, and cytokines that surround tumor cells and plays an essential role in tumor proliferation and immunotherapy sensitivity and resistance. The TME has been implicated across tumor types in both anti- and pro-tumorigenic pathways.

The degree of TILs has been associated with immunotherapy response, while T regulatory cells (Tregs) promote an immunosuppressive TME that allows for cancer immune evasion and growth. TILs in SCLC tend to be both fewer in number and seen at the stroma rather than the tumor core, contributing to a landscape of immune exclusion and contributing to their classification as “immunologically cold” tumors^[57]^. Patients who develop primary resistance demonstrate minimal or no T cell infiltration into the tumor, and there is evidence that some SCLC can secrete cytokines such as IL-10, which can drive differentiation of Tregs, leading to blunting of the antitumor immune response^[53,57,86]^. There is also evidence that TIL infiltration may be dynamic in the context of immunotherapy treatment in SCLC based on unpublished data from correlative studies performed on paired biopsies obtained at baseline, on treatment, and at progression in patients treated with ipilimumab/nivolumab on a Phase 2 trial at our institution. These patient samples demonstrated changes in TILs and MHC-I at different time points in treatment, with increases in CD8+ effector T cells, CD4+ helper T cells, and MHC-I during treatment, followed by a subsequent decline back to baseline levels upon disease progression. Certain subtypes of SCLC that were characterized as “neuroendocrine (NE)-high” have also been found to have decreased TIL infiltration compared to “NE-low” patient samples^[44]^.

Chemotactic cytokines (chemokines) play a significant role in immune cell migration and trafficking in tumors. IFN-γ inducible chemokines, including C-X-C motif ligand 9 (CXCL9), CXCL10, and CXCL11, have been associated with increased T-helper type 1 (Th1-type) immunity and antitumor response in multiple solid tumors, including melanoma, ovarian cancer, and squamous cell carcinoma^[87-90]^. CCL5 has been shown to be required for T cell infiltration and synergizes with CXCL9 to promote TIL infiltration, with high levels of these two chemokines associated with higher percentages of CD8+ and CD4+ T cells, as well as M1 macrophages^[88]^. Meanwhile, other cytokines, such as IL-10, TGF-β, and IL-35, lead to T cell inactivation and promote tumorigenesis^[91]^. While the cytotoxic CD8+ T cell is an essential component of the tumor-killing response, CD4+ T cell populations may support or hinder this process through the secretion of selected cytokines.

Although much of the work on immunotherapy resistance has been done on cells of the adaptive immune system, there is increasing evidence that cells of the innate immune system, including neutrophils and macrophages, also play an essential role in modulating the antitumor response. Myeloid-derived suppressor cells (MDSCs) include a heterogeneous population of immunosuppressive myeloid-lineage cells, and tumor recruitment of these cells has been associated with a worse prognosis^[92,93]^. Tumor-infiltrating neutrophils (TINs) and tumor-associated macrophages (TAMs) are also found in the milieu of immune cells that comprise the TME and have been found to create an immunosuppressed environment, which can promote tumor growth^[94,95]^. In addition, chemokines such as CCL7 and IL-8 recruit Tregs and myeloid-derived suppressor cells (MDSCs), while epigenetic modulators can silence chemokines involved in T cell migration, such as CXCL9 and CXCL10, to reduce TILs^[89]^. IDO1, for example, is an enzyme expressed in myeloid and cancer cells that converts tryptophan to an immunosuppressive metabolite, kynurenine, leading to decreased T cell clonal expansion^[96]^. Interestingly, there is evidence of higher levels of intra-tumoral IDO1 specifically in lymph nodes in the NE-low patient samples, which were densely infiltrated by immune cells; one hypothesis was that IDO overexpression was an escape mechanism in these patients that rendered TILs anergic, which is consistent with prior studies demonstrating an association between IDO1 and T cell inactivation^[44]^. Further supporting the role of the TME in immunotherapy response and the recognition of heterogeneity in SCLC, the recently identified SCLC-I-NE subtype is characterized by low TAM and high T-effector cells (lower TAM to T-effectors cell ratio) and demonstrates better outcomes with immunotherapy as compared to SCLC-I-nonNE, which displays higher immunosuppressive TAM levels^[43]^.

Upregulation of alternative immune checkpoint receptors including TIM-3, LAG-3, and TIGIT is also associated with T cell exhaustion/dysfunction^[97-99]^. Promising studies are underway to evaluate novel inhibitors for these alternative immune checkpoint receptors. Of note, however, the final PFS and OS analysis of the Phase 3 SKYSCRAPER-02 trial of tiragolumab, a TIGIT inhibitor, demonstrated no additional benefit with the addition of this drug to atezolizumab and platinum-etoposide^[100]^. These results suggest that additional blockade of these alternative immune checkpoints alone may not be sufficient and that the mechanism of resistance is more complex.

Other components of the TME, including the vasculature and the microbiome, have also been found to alter tumor response to ICIs through effects on immune cell populations^[101-104]^. Hypoxia and aberrant intratumoral vasculature are hallmarks of SCLC, with studies showing that hypoxia-inducible factor 1α (HIF-1α) is highly expressed in SCLC and elevated expression has been associated with a worse prognosis in these patients^[105-107]^. Hypoxia upregulates the expression of vascular endothelial growth factor (VEGF), an important pathway for increased tumor metastasis and growth, while aberrant vasculature can hinder ICI penetration as well as effector T cell trafficking and infiltration. In preclinical mouse models of SCLC, VEGFA could enhance the expression of the alternate inhibitory receptor TIM-3 in T cells from mice treated with anti-PD-L1 alone, leading to an exhausted T cell phenotype that was reversed with the addition of anti-VEGF^[108]^.

The association between baseline differences in vasculature and the immune system with ICI response has been validated in several solid tumor types, including melanoma and gastric cancer, and the combination of anti-VEGF agents and immunotherapy has seen positive results in preclinical models of SCLC^[101,108-110]^. Prior clinical studies of anti-VEGF agents had demonstrated mixed efficacy in SCLC patients until the recent publication of ETER701, a Phase 3 trial for first-line treatment of ES-SCLC comparing benmelstobart, anlotinib, and platinum-etoposide or anlotinib and platinum-etoposide to platinum-etoposide (the standard of care in China at the time of trial design)^[30,111-113]^. Benmelstobart is a monoclonal antibody targeting PD-1, while anlotinib is a small molecule multi-target tyrosine kinase inhibitor with anti-angiogenesis effects^[114,115]^. The quadruplet arm of benmelstobart, anlotinib, and platinum etoposide demonstrated an impressive OS (19.3 months versus 11.9 months, HR 0.61, 95% CI 0.47-0.79, *P* = 0.0002) and PFS benefit (6.9 months *vs.* 4.2 months, 95% CI 0.0.26-0.41, *P* < 0.0001), while the triple arm of anlotinib with platinum-etoposide did not demonstrate an OS benefit, lending support for the role of VEGF with immunotherapy in SCLC, though further molecular studies are still needed.

Finally, the gut microbiome has also become an area of interest for ICI response given the role of the microbiome in host immunity, with patients exposed to antibiotics before or during ICI administration associated with worse outcomes, though most studies are retrospective and there are certainly many confounding factors in these studies^[116]^. In one small prospective study, patients with SCLC treated with chemoimmunotherapy who were administered oral probiotics did demonstrate an improvement in mPFS (11.1 months *vs*. 7.0 months, *P* = 0.049) which does warrant further study^[117]^.

### Primary resistance *vs.* acquired resistance in SCLC

The mechanisms of primary and acquired resistance to immunotherapy likely overlap to some extent and involve both intrinsic and extrinsic mechanisms of resistance. Although research is currently ongoing, it is difficult to fully identify which are involved in each type of resistance at this time, in part because studies of mechanisms of acquired resistance have historically been difficult due to the paucity of tissue samples from the time of progression and issues with sufficient quality of sample for sequencing. Particularly compared to other solid tumors, there are few studies that have been able to examine these mechanisms in patient samples and very few paired biopsies in clinical trials.

Almost all patients with SCLC develop acquired resistance to first-line chemoimmunotherapy, with 90% of patients in the atezolizumab arm and 89% of patients in the durvalumab arm experiencing RECIST-defined disease progression at the time of updated 2-year analysis in IMpower133 and CASPIAN, respectively^[13,15]^. Several studies examining radiologic patterns of progression in SCLC have demonstrated that the most common sites of progression are in the primary lung lesion, lymph nodes, liver, and brain^[118]^.

Expansion of clonal populations may be a driver of acquired resistance. Subclonal expansion after cCRT in relapsed patients suggests that heterogeneous selection occurs, with the caveat that these were studied in patients who received chemoradiotherapy rather than chemoimmunotherapy or immunotherapy alone^[119]^ Heterogeneity within the tumor is likely a contributor, given evidence to suggest that even among MHC I-low or -negative tumors, clusters of tumor cells express high MHC I that may drive initial response^[45]^. Immune infiltration into primary versus metastatic sites can differ, with increased infiltration into liver or brain metastases compared to primary lung tumors^[120,121]^. Interestingly, brain metastases and adrenal metastases had higher TMB than lung tumors, suggesting that there may be differences in the immunogenicity of primary lung lesions compared to metastatic sites. This may form the basis for oligoprogression, wherein resistance develops in areas of low tumor immunogenicity. In addition, brain tumors were significantly enriched for PTEN alterations compared to lung or liver metastases, which has also been implicated in the evasion of the immune response^[58]^.

While it is certainly possible that oligoprogression occurs in a subclonal population, outcomes are mixed regarding the association of subtypes or the presence of heterogeneity in outcomes^[71,85]^. Dynamic changes in antigen presentation may also contribute to this response. Biopsies at the progression from a small cohort of patients treated with ipilimumab/nivolumab were found to have downregulation of β2M, suggesting a reduction in MHC I expression in response to immunotherapy^[122]^. One study found that patients at the time of acquired resistance to second-line ICI showed re-emergence of subclonal mutations that were present at diagnosis, prior to first-line chemotherapy^[123]^. These subclones were not seen prior to initiation of second-line ICIs and carried signs of platinum exposure from the patients’ first-line chemotherapies, suggesting that these cells expanded and seeded after ICI exposure. No specific mutations were shared among these patients to suggest a pathway for this re-emergence, although sample sizes were very small.

Subtype switching of molecular/transcriptomic subtypes has been hypothesized as a possible mechanism of resistance in SCLC, whereby non-neuroendocrine subtypes transition to neuroendocrine subtypes and vice versa. Although one study examined subtype switching after treatment, the cohort only included two patients treated with immunotherapy due to the timing of the study and neither patient demonstrated subtype switching^[124]^. Overall, our understanding of molecular SCLC subtypes and acquired resistance to immunotherapy is incomplete and will require concerted efforts to obtain and analyze samples at progression.

Several studies have analyzed transcriptomics in paired biopsies from patients who were treated with chemotherapy and have identified PI3K/AKT, HIF-1, MYC, and TGF-β pathways as potential mediators of acquired resistance, but similar studies in immunotherapy or chemoimmunotherapy are sparse in SCLC^[82,125,126]^.

### Immunotherapy in LS-SCLC

Consolidation durvalumab after concurrent chemoradiotherapy for LS-SCLC is already being adopted in clinical practice after inclusion in the NCCN guidelines, with the recent publication of the first interim analysis for ADRIATIC demonstrating significant PFS and OS benefit^[27]^. Of note, the addition of immunotherapy to concurrent chemoradiotherapy conferred a 22-month OS advantage for LS-SCLC, whereas, in ES-SCLC, the OS benefit is on the order of 2 months. At this time, it is unclear whether this difference represents differences in disease biology between limited-stage and advanced diseases or reflects the effects of radiation combined with immunotherapy.

It is not clear that the mutational landscape is significantly different between ES-SCLC and LS-SCLC. In one LS-SCLC cohort, the most frequently altered genes were *TP53* (91%), *RB1* (57%), *KMT2D* (18%), *PTPRT* (7%), *NOTCH1* (5%), and *STK11* (5%), which is similar to the alteration profiles seen in ES-SCLC^[127]^. Other studies have also remarked upon *SETDB2, FAM135B, LRP1B, ZFX4*, and *LRRK2* as mutations of interest in LS-SCLC. PD-L1 expression prevalence appears similarly low between limited-stage and extensive-stage disease^[52]^.

There is ample evidence that radiotherapy can induce tumor cell death, which increases the release of tumor antigens and could enrich immune activation-related pathways to enhance the effects of ICIs^[119,128]^. While this is a contributor, it is unlikely to be the sole driver of the difference in outcomes, as although consolidation radiation with immunotherapy in ES-SCLC has demonstrated efficacy, the improvement in outcomes is not nearly as dramatic^[129]^. With several other trials including consolidation immunotherapy or concurrent chemoimmunotherapy with radiotherapy, we anticipate that more data about these mechanisms of resistance will be forthcoming.

## CONCLUDING REMARKS

**Figure 5:**
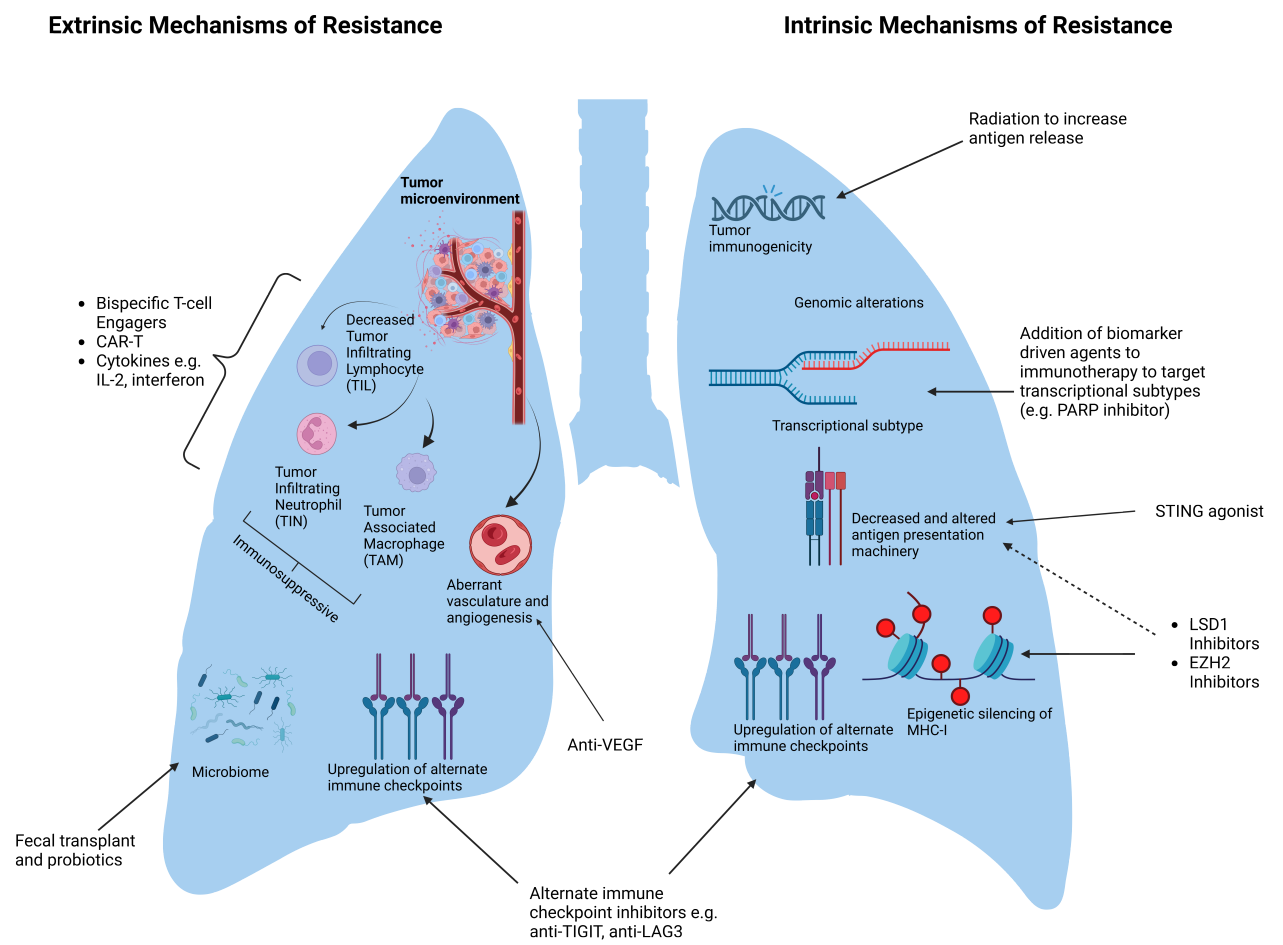
Summary of intrinsic and extrinsic mechanisms of resistance in SCLC and potential therapeutics to overcome resistance. Mechanism-directed strategies to overcome immunotherapy resistance and biomarker-driven therapies address the inherent heterogeneity in SCLC. The extrinsic mechanisms of resistance via interactions with the TME are comprised of a complex interplay of immune cells, vasculature, immune checkpoints, and microbiome. Therapies that may target aspects of the TME include cellular therapies, cytokine therapies, anti-angiogenesis agents, and immune checkpoint inhibitors. Intrinsic mechanisms of resistance, which include decreased tumor immunogenicity, altered antigen presentation machinery, immune checkpoints, and epigenetic modifications, may also be avenues for targeted therapies. SCLC: Small cell lung cancer; TME: Tumor microenvironment; CAR-T: Chimeric antigen receptor T-cell therapy; PARP: Poly-ADP ribose polymerase; STING: Stimulator of interferon genes.

A better understanding of the mechanisms of immunotherapy resistance will be essential for improving patient outcomes. With multiple therapeutics in development, the ability to predict which patients will derive benefit from immunotherapy alone will be critical for treatment sequencing. Meanwhile, a better understanding of the mechanisms of acquired resistance will drive the development of tailored treatment to bypass specific mechanisms, given the complexities of both extrinsic and intrinsic mechanisms of resistance [Figure 5]. As an example, bispecific T cell engagers can recruit T cells to the tumor if there are limited TILs and an “immune desert” is the driver of resistance. Epigenetic regulators such as EZH2 inhibition, LSD1 inhibition, and STING agonists represent research areas with the potential to overcome reduced MHC I expression. ADRIATIC suggests that the addition of radiation therapy to immunotherapy is well tolerated and improves outcomes, likely in the setting of release of tumor-associated antigens and increased immunogenicity. Although most patients with SCLC are diagnosed when the disease is advanced, there is a subset of LS-SCLC patients with node-negative disease for whom surgery may be an option. While perioperative and neoadjuvant immunotherapy has been studied in other solid tumors, large, randomized studies are still lacking for SCLC.

Given the historical dearth of biopsy samples in SCLC, particularly at the time of progression and outside of clinical trials, the common presence of extensive necrosis in tumor samples, and the paucity of human-relevant mouse models to test immunotherapy effects, understanding of these resistance mechanisms has lagged behind that of other solid tumors. Efforts are currently underway to better characterize and overcome these resistance mechanisms; however, many studies do not include paired biopsies, and tissue samples on treatment and at the time of progression are still relatively rare. An upcoming trial, S2409 PRISM (Multicohort PRecIsion SCLC Subtype Maintenance Phase II Trial of Durvalumab Versus Biomarker-Directed Novel Agents in Combination with Durvalumab in Extensive Stage Small Cell Lung Cancer), will require testing of archival tissue for eligibility. While this trial will be foundational in identifying predictive markers and novel agents to overcome immunotherapy resistance, paired biopsies and biopsies upon progression will remain essential to studying mechanisms of ICI response and acquired resistance.

## FUTURE DIRECTIONS

Overall, while patient selection for primary resistance can improve response rates, future directions should look toward methods to overcome resistance so that patients with SCLC can derive the durable benefit that immunotherapy brings. Given the recent advances in the treatment of SCLC and the widespread adoption of chemoimmunotherapy in the last 5 years, we are hopeful and expect significant progress in our understanding of and ability to bypass these resistance mechanisms.

## DECLARATIONS

### Copyright

© The Author(s) 2024.
